# Anti-Inflammatory and Antioxidant Properties of *Bauhinia thailandica* Leaf Extract in Microglial Cells

**DOI:** 10.3390/ijms27062809

**Published:** 2026-03-20

**Authors:** Wilawan Promprom, Wannachai Chatan, Kritsana Homwutthiwong, Kwanjit Apaijit, Poonlarp Cheepsunthorn, Nootchanat Mairuae

**Affiliations:** 1 Department of Biology, Faculty of Science, Mahasarakham University, Kantharawichai District, Maha Sarakham 44150, Thailand; wilawan.pp@msu.ac.th (W.P.); wannachaichatan@gmail.com (W.C.); 2 Biomedical Research Unit, Faculty of Medicine, Mahasarakham University, Maha Sarakham 44000, Thailand; kritsana.h@msu.ac.th (K.H.); kwanjit.s@msu.ac.th (K.A.); 3 Faculty of Medicine, Chulalongkorn University, Bangkok 10330, Thailand; poonlarp.c@chula.ac.th

**Keywords:** neuro-inflammation, oxidative stress, microglia, *Bauhinia thailandica*, total flavonoid contents, total phenolic contents, total tannin contents

## Abstract

Neuroinflammation is pivotal in the development of numerous neurodegenerative disorders, such as Alzheimer’s disease (AD), Parkinson’s disease (PD), and multiple sclerosis (MS). Microglial cells, the principal immune cells of the central nervous system (CNS), are essential mediators of this process. Upon exposure to pathogenic stimuli such as lipopolysaccharide (LPS), microglia activate and release pro-inflammatory mediators, leading to heightened oxidative stress and neuronal damage. Therefore, targeting microglial activation is a promising therapeutic approach to prevent or slow neurodegeneration. This study aimed to investigate the antioxidant and anti-inflammatory effects of the leaf extract of the newly identified species *Bauhinia thailandica* on LPS-activated BV2 microglia. The phytochemical compound of the *B. thailandica* leaf extract was also investigated. BV2 cells were treated with LPS (1 μg/mL) for 24 h in the presence or absence of *B. thailandica* leaf extract (12.5 and 25 µg/mL). The levels of reactive oxygen species (ROS), nitric oxide (NO), and interleukin-6 (IL-6), IL-1β, and tumor necrosis factor-alpha (TNF-α) were quantified with CM-H2DCFDA, Griess reagent assay, and ELISA, respectively. Treatment with LPS resulted in significant increases in ROS, NO, IL-6, IL-1, and TNF levels compared to untreated cells (*p* < 0.01). However, co-treatment with *B. thailandica* leaf extract significantly suppressed the production of these inflammatory markers (*p* < 0.01 for 25 µg/mL across all parameters, except TNF-α; *p* < 0.05). The results also showed that *B. thailandica* leaf extract possessed significant levels of total phenolic content (TPC; 70.55 mg GAE/g dry extract), total flavonoid content (TFC; 249.47 mg QE/g dry extract), and tannins (397.50 mg TAE/g dry extract). Phytochemical screening also revealed the presence of saponins and cardiac glycosides in the extract. In conclusion, the leaf extract of *B. thailandica* is a potent source of phytochemicals exhibiting antioxidant capabilities and has shown both antioxidant and anti-inflammatory actions in LPS-activated BV2 microglial cells. The findings indicate that *B. thailandica* leaf extract shows significant promise as a novel herbal treatment for neuroinflammatory disorders mediated by microglia. Further research is necessary to clarify the underlying mechanisms of action and to investigate the active substances responsible for these effects.

## 1. Introduction

Microglial cells are acknowledged as resident macrophages in the brain, important for sustaining neuroprotective activities and homeostasis through continuous monitoring and removal of debris [1,2,3]. They serve as the primary defense of the neuroimmune system, influencing synaptic plasticity, metabolic regulation inside the brain, and the modulation of learning and memory [1,4]. Microglia are activated by neurotoxic stimuli, genetic changes, or other harmful events. This activation is very important for neuroinflammation and exacerbates neurodegenerative illnesses such as Alzheimer’s disease (AD), Parkinson’s disease (PD), and multiple sclerosis (MS) [5,6]. Neuroinflammation, mostly induced by activated microglia, results in the excessive synthesis of pro-inflammatory cytokines, including tumor necrosis factor-alpha (TNF-α), interleukin-1β (IL-1β), and interleukin-6 (IL-6), hence exacerbating neuronal damage [3,7]. Additionally, multiple studies have shown that microglial activation causes neuroinflammation and plays a key role in the onset of depression, dementia, and cognitive impairment [8,9,10,11]. AD primarily affects older adults and is significantly correlated with age-related microglial activation [12,13]. Research on the isolation of microglia from young and aged mice demonstrated that aged microglia exhibit heightened basal production of IL-6 and TNF-α, reduced glutathione levels, and impaired amyloid-β clearance [14,15,16]. These findings indicate that aging enhances microglial inflammatory responses, thereby promoting neuroinflammation, neurodegeneration, and cognitive decline. Additionally, microglial reactivity to interferon-γ (IFN-γ) escalates with aging, promoting a transition to a pro-inflammatory M1-like phenotype, hence diminishing the neuroprotective M2 phenotype [10,17]. As a result, treatment approaches designed to impede microglial activation or avert the M2-to-M1 phenotypic shift may function as efficacious neuroprotective measures [3,18].

Current pharmacological interventions often exhibit limited efficacy and may cause adverse effects. As a result, natural bioactive compounds have garnered increasing attention as prospective alternative therapeutic agents for alleviating neuroinflammation and oxidative stress [11,19,20,21,22,23]. Numerous plant-derived secondary metabolites, including flavonoids, phenolic acids, tannins, alkaloids, and terpenoids, have demonstrated neuroprotective properties. These compounds are useful because they neutralize free radicals, modulate inflammatory pathways, reduce microglial activation, and improve cellular antioxidant defense systems [24]. Importantly, different groups of phytochemicals have unique structures that shape their biological effects. Flavonoids possess a polyphenolic backbone with specific hydroxyl groups that contribute to their strong antioxidant properties. These compounds are known to scavenge reactive oxygen species (ROS) and regulate key inflammatory signaling pathways, including nuclear factor kappa B (NF-κB), mitogen-activated protein kinase (MAPK), and the nuclear factor erythroid 2-related factor 2 (Nrf2) antioxidant response [25,26]. Tannins are another type of large polyphenols. They have strong antioxidant effects and can lower the production of inflammatory enzymes and cytokines [27]. Other bioactive groups, such as alkaloids and terpenoids, affect signaling pathways involved in immune responses and oxidative stress [28]. Understanding these bioactive class/function correlations provides a molecular basis for selecting plant species with potential neuroprotective properties.

*Bauhinia* L. is a genus of the family Leguminosae (Fabaceae) and is currently classified under the subfamily Cercidoideae according to modern phylogeny-based classification of legumes [29]. In the present study, we follow the currently accepted classification and consistently treat *Bauhinia* as a member of Cercidoideae. Species of *Bauhinia* are widely distributed in tropical and subtropical regions and are valued as ornamental, medicinal, and ethnobotanically important plants [29]. Previous phytochemical studies have reported that *Bauhinia* species contain diverse secondary metabolites, including phenolics, flavonoids, tannins, terpenoids, steroids, alkaloids, quinones, lactones, and aromatic acids. These phytochemicals are believed to contribute to their antioxidant, anti-inflammatory [30], and other pharmacological activities [31]. The documented abundance of flavonoids and phenolic compounds in this genus supports its potential relevance in modulating oxidative stress and inflammatory signaling.

*Bauhinia thailandica* Chatan & Promprom (Siao-Phum) is a newly identified endemic species from Thailand, classified within the subfamily Cercidoideae of the Leguminosae family [32]. It was first discovered in 2018. Phu Pha Yon National Park in Sakon Nakhon Province, Thailand, is currently the only known habitat for this species. It grows in dry deciduous forests and along roadsides in dry evergreen forests at elevations of 300 to 600 m. Several species within the genus *Bauhinia* have been reported to contain bioactive compounds with antioxidant and anti-inflammatory properties. In contrast, *B*. *thailandica* is a newly described species, and information regarding its phytochemical composition and biological activities remains limited. Considering the phytochemical diversity observed in related *Bauhinia* species, especially the presence of flavonoids and phenolic compounds with known anti-inflammatory and antioxidant effects, *B. thailandica* is a promising candidate for research into microglia-mediated neuroinflammation.

The objective of this study was to analyze the phytochemical composition of *B. thailandica* leaf extract and evaluate its anti-inflammatory and antioxidant effects in BV2 microglial cells activated by lipopolysaccharide (LPS). This study focused on the extract’s capacity to modulate ROS generation, nitric oxide (NO) production, and pro-inflammatory cytokine expression. This research seeks to provide mechanistic insights into the neuroprotective potential of *B. thailandica* and support the identification of novel plant-derived compounds targeting microglia-driven neuroinflammation. LPS was selected as a stimulant because it is widely used to activate microglia in experimental models and mimics bacterial endotoxin exposure. It reliably induces inflammatory responses through activation of the Toll-like receptor 4 (TLR4) signaling pathway [33]. This results in increased production of pro-inflammatory cytokines, nitric oxide, and ROS [33].

## 2. Results

### 2.1. Phytochemical Screening

Qualitative phytochemical analysis of *Bauhinia thailandica* leaf extract indicated the existence of several bioactive chemicals. Table 1 shows that the extract tested positive for phenolics, flavonoids, tannins, cardiac glycosides, and saponins. This was shown by color changes that are typical of conventional testing. However, terpenoids, steroids, alkaloids, and anthraquinones were not identified. These results indicate that *B. thailandica* leaves are abundant in polyphenolic and glycosidic chemicals, potentially enhancing their antioxidant properties [34].

### 2.2. Quantitative Analysis of Total Phenolic, Flavonoid, and Tannin Contents

The quantitative analysis of major phytochemical constituents in the ethanolic leaf extract of *B. thailandica* is presented in Table 2. The extract contained 70.55 ± 1.35 mg GAE/g dry extract of total phenolics, 249.47 ± 1.24 mg QE/g dry extract of total flavonoids, and 397.50 ± 4.44 mg TAE/g dry extract of total tannins. It should be noted that these values were determined using different colorimetric assays and expressed as calibration equivalents: gallic acid equivalent (GAE), quercetin equivalent (QE), and tannic acid equivalent (TAE). Therefore, these results should be interpreted as assay-specific equivalent values rather than directly comparable absolute concentrations. Nevertheless, the results indicate the presence of notable amounts of phenolic-related constituents in the extract, which may contribute to its antioxidant and other biological properties [35].

### 2.3. DPPH Radical Scavenging Activity

The antioxidant activity of the *B. thailandica* leaf extract was evaluated using the DPPH radical scavenging assay. The extract demonstrated a concentration-dependent increase in radical scavenging activity, reaching 89.90 ± 1.36% inhibition at 1000 µg/mL (Table 3). The IC_50_ value of the extract was 513.60 ± 7.20 µg/mL. Vitamin C, used as the positive control, showed 80.91 ± 0.42% inhibition at 100 µg/mL and an IC_50_ of 68.25 ± 2.31 µg/mL. Based on the lower IC_50_ value, vitamin C exhibited stronger DPPH radical scavenging activity than the extract [36,37].

### 2.4. Impact of B. thailandica Leaf Extract on Cellular Viability

**Figure 1:**
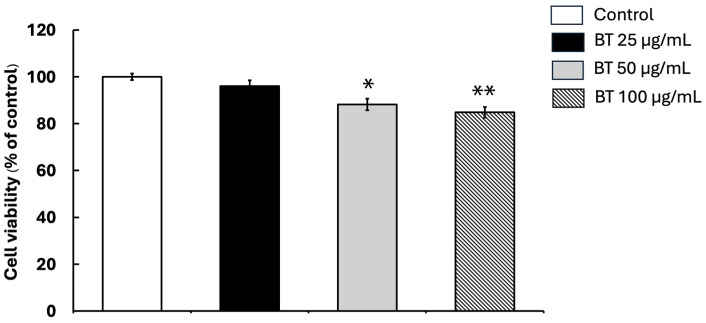
The impact of *B. thailandica* leaf extract on the survival of BV2 microglial cells. The data are shown as the mean ± SEM of three separate experiments. * *p* < 0.05, ** *p* < 0.01 in comparison to the control group. BT: *B. thailandica*.

An MTT test was used to assess whether *B. thailandica* leaf extract is toxic to BV2 microglial cells. This colorimetric assay measures cell viability by assessing mitochondrial activity, which helps assess cell health after cells are exposed to different doses of the extract. The results show that treatment with *B. thailandica* leaf extract for 24 h at concentrations up to 25 µg/mL had no cytotoxic effects on BV2 cells (Figure 1). Cell viability remained high across all tested concentrations. The control group was set as 100%, while cell viability values were 95.98% ± 2.50, 88.16% ± 2.28, and 84.90% ± 2.26 at the tested extract concentrations (Figure 1). The results also show that BV2 cells tolerated the extract well under the test conditions. Based on this, we selected two non-cytotoxic concentrations, 12.5 µg/mL and 25 µg/mL, for further experiments to investigate its potential antioxidant and anti-inflammatory effects in BV2 microglial cells.

### 2.5. Impact of B. thailandica Leaf Extract on ROS Generation

**Figure 2:**
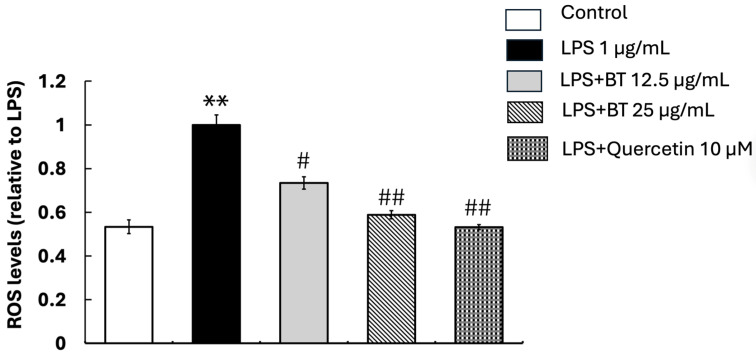
Intracellular ROS levels in BV2 microglial cells following LPS stimulation and treatment with *B*. *thailandica* leaf extract. ROS levels were normalized to the LPS-treated group (set as 1). Data are presented as the mean ± SEM of three independent experiments. ** *p* < 0.01, compared with the control group; # *p* < 0.05, compared with the LPS-treated group; ## *p* < 0.01, compared with the LPS-treated group. BT: *B. thailandica*; LPS: lipopolysaccharide.

We tested the antioxidant effect of *B. thailandica* leaf extract by detecting ROS levels inside cells during microglial activation. The generation of ROS is a significant marker of oxidative stress, which plays a pivotal role in neurodegenerative disorders and neuroinflammation. Further supporting the production of oxidative stress, BV2 microglial cells exposed to LPS showed a significant increase in ROS production after 24 h compared with the control group (*p* < 0.01) (Figure 2). ROS levels in the LPS-treated group were normalized to 1. Co-treatment with *B*. *thailandica* leaf extract significantly reduced ROS levels compared with the LPS group in a concentration-dependent manner. ROS levels decreased to 0.73-fold and 0.59-fold relative to LPS-treated cells following treatment with 12.5 and 25 µg/mL, respectively (*p* < 0.05 and *p* < 0.01). The quercetin-treated group, used as a positive control, also showed a significant reduction in ROS levels, confirming the assay validity. These results indicate that *B. thailandica* leaf extract possesses robust antioxidant properties that effectively mitigate oxidative stress in activated microglial cells. This suggests that it may possess therapeutic potential in conditions associated with neuroinflammation and oxidative injury.

### 2.6. Impact of B. thailandica Leaf Extract on LPS-Induced Nitric Oxide Synthesis

**Figure 3:**
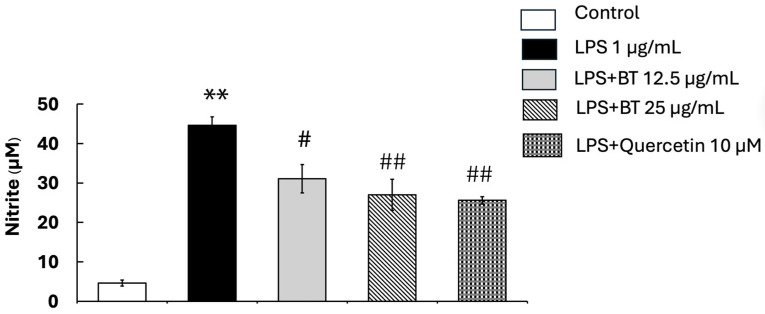
Effect of *B. thailandica* leaf on LPS-induced NO production in BV2 microglial cells. Data are presented as the mean ± SEM of three independent experiments. ** *p* < 0.01, compared with the control group; # *p* < 0.05, compared with the LPS-treated group; ## *p* < 0.01, compared with the LPS-treated group. BT: *B. thailandica*; LPS: lipopolysaccharide.

To evaluate the anti-inflammatory efficacy of *B*. *thailandica* leaf extract, its impact on NO synthesis in LPS-activated BV2 microglial cells was examined. Figure 3 demonstrates that LPS stimulation markedly elevated NO levels in BV2 cells after 24 h of treatment (44.6 ± 2.19 μM) compared with the untreated control group (4.6 ± 0.74 μM) (*p* < 0.01), indicating effective microglial activation. Co-treatment with *B*. *thailandica* leaf extract markedly suppressed LPS-induced NO generation in a concentration-dependent manner. After treatment with 12.5 μg/mL and 25 μg/mL of the extract, NO levels dropped to 31.1 ± 3.58 μM and 27.0 ± 3.93 μM, respectively. A statistically significant reduction (*p* < 0.05 and *p* < 0.01) was observed at the concentrations of 12.5 μg/mL and 25 μg/mL, indicating a considerable anti-inflammatory effect. Quercetin, employed as a positive control, markedly reduced NO generation to 25.6 ± 0.91 μM, compared with the LPS-treated group (*p* < 0.01). These results demonstrate that *B*. *thailandica* leaf extract efficiently inhibits NO-mediated inflammatory responses in activated microglial cells and may confer protective effects against neuroinflammation.

### 2.7. Impact of B. thailandica Leaf Extract on LPS-Induced IL-6 Synthesis

**Figure 4:**
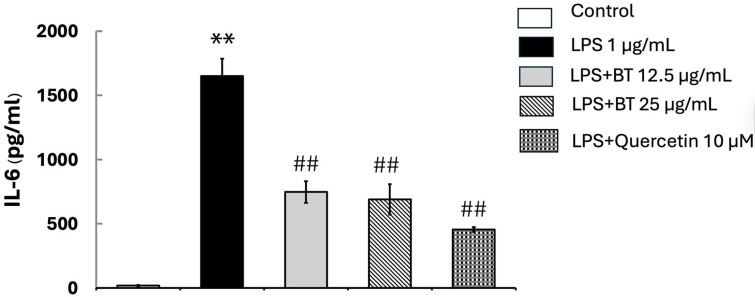
Effect of *B. thailandica* leaf extract on LPS-induced IL-6 production. Data are shown as the mean ± SEM of three independent experiments. ** *p* < 0.01, compared with the control group; ## *p* < 0.01, compared with the LPS-treated group. BT: *B. thailandica*; LPS: lipopolysaccharide.

ELISA was conducted to assess the impact of *B. thailandica* leaf extract on the production of IL-6 in LPS-activated BV2 microglial cells. After 24 h of treatment, IL-6 levels were markedly elevated in LPS-treated cells (1650 ± 135.02 pg/mL) compared to the untreated control (17.5 ± 5.82 pg/mL) (*p* < 0.001; Figure 4). The production of IL-6 was substantially reduced in a concentration-dependent manner using co-treatment with *B. thailandica* leaf extract. Following treatment with 12.5 μg/mL and 25 μg/mL of the extract, IL-6 levels decreased to 747.25 ± 83.45 pg/mL and 689.5 ± 119.08 pg/mL, respectively. Similarly, quercetin significantly decreased IL-6 levels (454.75 ± 57.18 pg/mL) in comparison to the LPS group (*p* < 0.01). These results suggest that *B. thailandica* leaf extract has substantial anti-inflammatory efficacy by suppressing IL-6 production in activated microglial cells.

### 2.8. Impact of B. thailandica Leaf Extract on LPS-Induced IL-1β Synthesis

**Figure 5:**
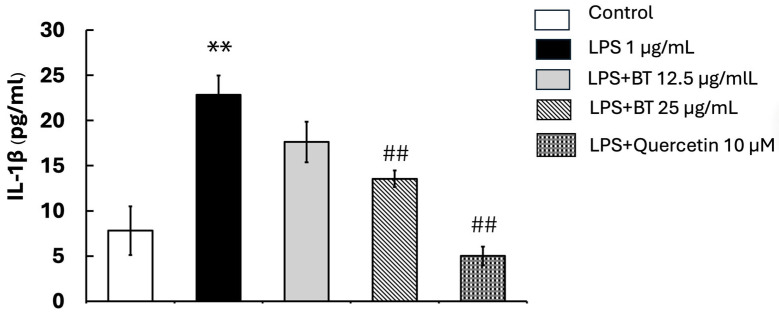
Effect of *B. thailandica* leaf extract on LPS-induced 1β production. Data are shown as the mean ± SEM of three independent experiments. ** *p* < 0.01, compared with the control group; ## *p* < 0.01, compared with the LPS-treated group. BT: *B. thailandica*; LPS: lipopolysaccharide.

To evaluate the anti-inflammatory effects of *B. thailandica* leaf extract, the production of IL-1β in LPS-stimulated BV2 microglial cells was evaluated using ELISA. As shown in Figure 5, the LPS-only treatment significantly increased IL-1β levels in the culture medium (22.85 ± 2.15) after 24 h of treatment, compared to the untreated control group (7.81 ± 2.69). This suggests a robust inflammatory response. Nevertheless, the production of IL-1β in BV2 cells was suppressed in a concentration-dependent manner using co-treatment with *B. thailandica* leaf extract (17.62 ± 2.25 and 13.55 ± 0.91 for 12.5 and 25 μg/mL, respectively). Additionally, the quercetin treatment resulted in a substantial reduction in IL-1β levels compared to the LPS-treated group (5.01 ± 1.03; *p* < 0.01). These results suggest that *B. thailandica* leaf extract possesses potent anti-inflammatory properties, achieved by modulating cytokine signaling pathways. The extract’s potential as a natural therapeutic agent for controlling neuroinflammation and preventing microglial overactivation in neurodegenerative conditions is further supported by its ability to suppress IL-1β production.

### 2.9. Impact of B. thailandica Leaf Extract on LPS-Induced TNF-α Synthesis

**Figure 6:**
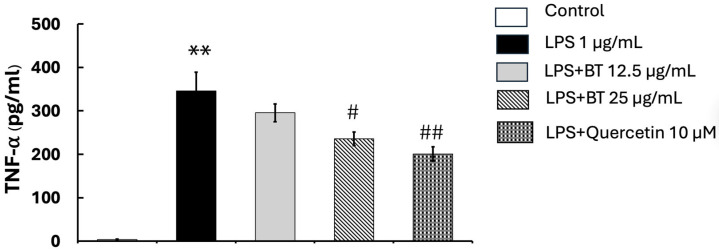
Effect of *B. thailandica* leaf extract on LPS-induced TNF-α production. Data are shown as the mean ± SEM of three independent experiments. ** *p* < 0.01, compared with the control group; # *p* < 0.05, ## *p* < 0.01, compared with the LPS-treated group. BT: *B. thailandica*; LPS: lipopolysaccharide.

We used an ELISA kit to assess how *B. thailandica* leaf extract affected TNF-α production in LPS-activated BV2 microglial cells, to assess its anti-inflammatory activity. As shown in Figure 6, stimulation with LPS alone markedly increased TNF-α levels in the culture medium after 24 h (345.66 ± 43.22) compared with the untreated control group (3.33 ± 1.26). This confirms successful induction of an inflammatory response. However, the concentration-dependent co-treatment with *B. thailandica* leaf extract decreased the generation of TNF-α in BV2 cells (295.33 ± 20.52 and 235.5 ± 15.55 for 12.5 and 25 μg/mL, respectively). This means that the extract works well to reduce inflammation by decreasing TNF-α production. These findings indicate that *B. thailandica* leaf extract possesses significant anti-inflammatory effects. Moreover, the quercetin administration resulted in a substantial reduction in TNF-α levels (200.83 ± 16.26) relative to the LPS-treated group (*p* < 0.01).

## 3. Discussion

The present study aimed to investigate whether *Bauhinia thailandica* leaf extract could attenuate oxidative stress and inflammatory responses in LPS-stimulated BV2 microglial cells. Our findings demonstrate that *B. thailandica* leaf extract markedly inhibited LPS-induced oxidative and inflammatory responses. This inhibitory effect was associated with decreased intracellular ROS, NO, and pro-inflammatory cytokines, including TNF-α, IL-1β, and IL-6. In addition, cytotoxicity analysis showed that the extract was well tolerated by BV2 cells at concentrations up to 25 µg/mL, indicating that the observed inhibitory effects were not associated with cytotoxicity. These findings suggest that *B. thailandica* leaf extract effectively modulates microglial activation and may exert protective effects against neuroinflammation. These results support the potential of this plant as a source of bioactive compounds capable of regulating neuroinflammatory processes.

Oxidative stress plays a critical role in the progression of neurodegenerative diseases, including AD, PD, and ALS [9,11,38,39]. Activated microglia produce excessive levels of ROS and inflammatory mediators, which contribute to neuronal injury and neurodegeneration [18,40,41,42,43,44,45]. In the present study, LPS stimulation significantly increased ROS production in BV2 microglial cells, confirming the induction of oxidative stress. However, co-treatment with *B. thailandica* leaf extract markedly reduced intracellular ROS levels in a dose-dependent manner. This effect was comparable to that of quercetin, a well-known antioxidant flavonoid [46], suggesting that the extract possesses strong antioxidant capacity capable of restoring cellular redox balance. This similarity highlights the prospective potential of *B. thailandica* leaf extract as a natural source of bioactive chemicals with antioxidant properties. The reduction in intracellular ROS may also contribute to the suppression of downstream inflammatory signaling pathways involved in microglial activation.

In addition to suppressing oxidative stress, *B. thailandica* leaf extract also significantly inhibited the production of inflammatory mediators. LPS stimulation markedly increased NO levels and the secretion of pro-inflammatory cytokines (IL-6, IL-1β, and TNF-α). Excessive NO production by activated microglia contributes to neuronal damage through the formation of peroxynitrite, a potent oxidant [44,47]. In the present study, treatment with *B. thailandica* leaf extract significantly reduced NO production in LPS-stimulated BV2 cells. Furthermore, ELISA analysis demonstrated that the extract markedly suppressed the release of IL-6, IL-1β, and TNF-α. TNF-α and IL-1β are key pro-inflammatory cytokines that amplify neuroinflammatory signaling cascades by activating multiple intracellular pathways, including NF-κB, JAK/STAT, and MAPK [48]. These cytokines are primarily produced by activated microglia and astrocytes in response to injury or infection, leading to the release of additional inflammatory mediators that sustain and escalate neuroinflammation [48]. Their inhibition suggests that the extract can disrupt the self-propagating inflammatory responses associated with chronic microglial activation. Collectively, these findings indicate that the extract exerts anti-inflammatory effects by attenuating both oxidative stress and cytokine-mediated inflammatory signaling.

Phytochemical screening of *B. thailandica* leaf extract revealed the presence of phenolics, flavonoids, tannins, saponins, and cardiac glycosides. These classes of phytochemicals are widely recognized for their antioxidant and anti-inflammatory activities and have been reported to modulate key signaling pathways involved in microglial activation [25,26]. Polyphenolic compounds, particularly flavonoids and tannins, are known to scavenge reactive oxygen species and suppress inflammatory signaling pathways [25,26,27,49,50,51]. Flavonoids also exhibit strong antioxidant activity through structural features that enable them to neutralize lipid peroxyl radicals, hydroxyl radicals, and superoxide anions [52]. Moreover, several flavonoids have been reported to inhibit microglial overactivation by regulating major inflammatory pathways, including NF-κB and MAPK, while activating the Nrf2-mediated antioxidant defense system [25,26]. In addition to polyphenols, other phytochemicals may also contribute to the observed biological activities. Saponins have been shown to reduce intracellular ROS levels and enhance endogenous antioxidant enzyme activities such as superoxide dismutase and catalase [53,54]. They can also suppress NO production through inhibition of NF-κB-mediated inflammatory signaling in activated immune cells [55]. Cardiac glycosides have likewise been reported to exert anti-inflammatory effects by modulating cytokine production and immune signaling pathways [56]. Tannins are powerful antioxidants because they donate hydrogen from their phenolic hydroxyl groups, which keeps free radicals stable. They have also been shown to lower the levels of pro-inflammatory substances such as TNF-α, IL-1β, IL-6, and inducible nitric oxide synthase [50,57,58].

Taken together, the presence of phenolics, flavonoids, tannins, saponins, and cardiac glycosides in *B. thailandica* leaf extract may contribute to the antioxidant and anti-inflammatory activities observed in LPS-activated BV2 microglial cells. These phytochemical constituents have been reported to regulate oxidative stress and inflammatory responses through pathways associated with microglial activation, including NF-κB, MAPK, and the Nrf2-mediated antioxidant defense system [25,26]. Such mechanisms may partially explain the reductions in intracellular ROS, NO, and pro-inflammatory cytokines observed in this study. Therefore, the inhibitory effects of *B. thailandica* leaf extract on microglial activation may be attributed, at least in part, to the combined actions of these bioactive phytochemicals.

Further research is necessary to identify and characterize the active constituents of *B. thailandica* leaf extract using chromatographic profiling techniques such as high-performance liquid chromatography (HPLC). Additionally, studies should elucidate the molecular mechanisms responsible for its neuroprotective effects. Future investigations are encouraged to determine whether the extract modulates key signaling pathways associated with microglial activation and neuroinflammation, including the NF-κB, MAPK, and Nrf2/HO-1 pathways. These studies would enhance understanding of the mechanistic basis for the observed antioxidant and anti-inflammatory activities and could support the development of *B. thailandica* as a potential source of therapeutic agents for neuroinflammatory and neurodegenerative disorders.

## 4. Materials and Methods

### 4.1. Chemicals and Reagents

All cell culture reagents, including penicillin/streptomycin, were sourced from Hyclone (Logan, UT, USA). The ROS detection kit and MTT assay kit were acquired from Sigma-Aldrich (St. Louis, MO, USA). ELISA kits were purchased from ABclonal Technology Co., Ltd. (Woburn, MA, USA).

### 4.2. Preparation of Bauhinia thailandica Leaf Extract

Leaves of *B*. *thailandica* were dried by microwave-assisted hot-air drying at 50 °C and 1000 W for 2 h, finely ground using a mechanical grinder, and sieved through an 80-mesh sieve to obtain a homogeneous powder. A 100 g portion of the powdered material was extracted with 95% ethanol at a solid-to-solvent ratio of 1:10 (*w*/*v*). Ultrasound-assisted extraction was carried out at 20 kHz and 50 °C for 30 min per cycle for a total of three cycles. After each cycle, the extract was filtered through Whatman No. 1 filter paper, and the residue was re-extracted under the same conditions. The combined filtrates were concentrated under reduced pressure using a rotary evaporator at 40 °C to obtain the crude ethanolic extract. The extraction yield of the crude extract was 15.67%.

### 4.3. Phytochemical Analysis (Qualitative)

Qualitative phytochemical analysis of the *B. thailandica* leaf extract was performed using standard screening methods, with minor modifications, to detect the major classes of secondary metabolites [52]. In each assay, 1 mL of the extract solution was used, and all tests were performed in triplicate using freshly prepared reagents [59]. Phenolic compounds were detected by the ferric chloride test using three drops of 10% FeCl_3_ solution, with the appearance of a dark blue, bluish-green, or blackish coloration taken as a positive result. Flavonoids were examined using the ammonia test by mixing the extract with 1 mL of 1% AlCl_3_ and 2 mL of 10% NH_3_, then adding 5 drops of concentrated H_2_SO_4_; a yellow coloration indicated a positive reaction. Tannins were identified by the ferric chloride test using three drops of 1% FeCl_3_ solution, with a blue-black or greenish-black coloration considered positive. Cardiac glycosides were screened using the Keller–Killiani test by adding 2 mL of glacial acetic acid containing 1 drop of 5% FeCl_3_, then carefully layering 1 mL of concentrated H_2_SO_4_; the formation of a brown ring at the interface indicated a positive result. Saponins were evaluated using the frothing test, in which the extract solution was diluted with distilled water and shaken vigorously for 1–2 min; the formation of a stable froth was considered positive. Terpenoids were assessed using the Salkowski test by mixing the extract with 1 mL of chloroform, followed by 0.5 mL of concentrated H_2_SO_4_; a reddish-brown coloration at the interface indicated the presence of terpenoids. Steroids were also examined using the Salkowski reaction under the same conditions, and a yellowish-green coloration or fluorescence in the acid layer was considered positive. Alkaloids were determined using Dragendorff’s test by acidifying the extract with 1 mL of 2% H_2_SO_4,_ followed by the addition of three drops of Dragendorff’s reagent; the formation of an orange or reddish-brown precipitate indicated the presence of alkaloids. Anthraquinones were analyzed using Borntrager’s test by mixing the extract with 1 mL of 10% H_2_SO_4_ and 0.5 mL of 10% NH_3_, with the appearance of a pink to red coloration taken as a positive reaction. The results were recorded as positive (+) or negative (−) according to the presence or absence of the characteristic reaction observed in each assay.

### 4.4. Estimation of Total Phenolic, Flavonoid, and Tannin Contents

Total phenolic content was determined using the Folin–Ciocâlteu method. Briefly, 0.5 mL of plant extract was mixed with 2.5 mL of 10% Folin–Ciocâlteu reagent and incubated for 5 min. Then, 2 mL of 7.5% sodium carbonate solution was added, and the mixture was left in the dark at room temperature for 30 min. Absorbance was measured at 765 nm. The results were expressed as milligrams of gallic acid equivalent per gram of dry extract (mg GAE/g dry extract).

Total flavonoid content was measured using the aluminum chloride colorimetric method. A 0.5 mL extract was mixed with 0.1 mL of 10% aluminum chloride, 0.1 mL of 1 M potassium acetate, and 4.3 mL of distilled water. After 30 min at room temperature, absorbance was recorded at 415 nm. The results were reported as milligrams of quercetin equivalent per gram dry extract (mg QE/g dry extract).

Total tannin content was evaluated using the Folin–Denis method. One milliliter of extract was mixed with 5 mL of Folin–Denis reagent and 10 mL of 7.5% sodium carbonate. The mixture was incubated at room temperature for 30 min, and absorbance was measured at 700 nm. Tannin content was expressed as milligrams of tannic acid equivalent per gram of dry extract (mg TAE/g dry extract) [60].

### 4.5. DPPH Radical Scavenging Method

The antioxidant activity of *B. thailandica* leaf extract was evaluated using the DPPH (2,2-diphenyl-1-picrylhydrazyl) free radical scavenging assay. A 1 mL aliquot of extract at various concentrations was mixed with 1 mL of 0.1 mM DPPH solution in methanol. The mixture was incubated in the dark at room temperature for 30 min. Absorbance was measured at 517 nm using a UV-Vis spectrophotometer (GENESYS 150, Thermo Fisher Scientific, Waltham, MA, USA). Methanol was used as a blank, and vitamin C served as the positive control. The percentage of radical scavenging activity was calculated using the following formula:

% Inhibition = [(A_0_ − A_1_)/A_0_] × 100

where A_0_ is the absorbance of the control and A_1_ is the absorbance of the sample. The IC_50_ value, defined as the concentration required to inhibit 50% of DPPH radicals, was determined from the dose–response curve. All measurements were conducted in triplicate.

### 4.6. Cell Cultures and Treatments

Murine BV2 microglial cells were kind gifts from Dr. James R. Connor of the Department of Neurosurgery, Pennsylvania State University College of Medicine, Hershey, PA. The cells were maintained in Dulbecco’s modified Eagle’s medium (DMEM) supplemented with 5% fetal bovine serum (FBS), 2 mM L-glutamine, 100 µg/mL streptomycin, and 100 U/mL penicillin. The cultures were incubated at 37 °C in a humidified atmosphere containing 5% CO_2_. The culture medium was replaced twice weekly while the cells were growing. When the cells reached approximately 80% confluence, they were subcultured for further maintenance. For experimental assays, cells were plated at a density of 1 × 10^4^ cells/well in 96-well plates and allowed to attach overnight before treatments were initiated. After overnight, the medium in each well was completely removed. In the assay of cell viability, the cells in each well were gently washed once with serum-free DMEM before adding freshly prepared serum-free DMEM containing specified concentrations of *B. thailandica* leaf extract ranging from 0 to 100 μg/mL in the presence or absence of LPS at a concentration of 1 µg/mL. Prior to the determination of ROS, NO, IL-6, IL-1β, and TNF-α levels, the growth medium in each well was completely removed and replaced with a medium containing LPS, with or without the specified concentrations of *B. thailandica* leaf extract. Cells in serum-free DMEM functioned as the untreated control. Serum-free conditions were maintained during the entire treatment period to avert any potential interference from serum-derived growth factors and cytokines that might affect LPS-induced inflammatory signaling and nitric oxide production. This method is often used in short-term inflammatory stimulation experiments to make sure that the effects that were reported are only because of LPS and the extract that was tested. The treatment lasted only 24 h, and the MTT test was used to check the status of the cells. The results showed that neither LPS nor the extract had a large effect on the viability of BV2 cells when there was no serum present at the doses that were tested. As a result, the serum-free environment did not induce significant cellular stress, which might have complicated the interpretation of the results [61,62].

### 4.7. Cell Viability Assay

After 24 h of treatment, the medium was removed from each well and replaced with 0.1 mL of MTT reagent (0.4 mg/mL; Sigma Co., St. Louis, MO, USA) in serum-free DMEM, then incubated at 37 °C for 2 h. After the incubation period, the MTT solution was removed from each well, and dimethyl sulfoxide was added to solubilize the formazan crystals. After each well was placed on a microplate mixer for 10 min, the optical density (OD) of the formazan solution was read at 570 nm using a plate reader (Spectramax 340 PC, Molecular Devices, San Jose, CA, USA) [61]. Cell viability (%) was calculated as (Absorbance of treated cells/Absorbance of control cells) × 100, with the control group set as 100% viability.

### 4.8. Nitric Oxide (NO) Assay

The levels of NO were determined by measuring nitrite accumulation in the cell culture supernatants using Griess reagent [1% sulfanilamide/0.1% N-(1-naphthyl)-ethylenediamine dihydrochloride 2.5% phosphoric acid] (Invitrogen, Carlsbad, CA, USA). In this assay, the nitrite-containing sample (150 µL/well) was placed in a 96-well plate, mixed with 20 µL of Griess reagent and 130 µL of deionized water, and incubated for 30 min at room temperature. Absorbance values were read at 540 nm using a microplate reader (Bio-Tek Instruments Inc., Winooski, VT, USA) [62]. The concentrations of nitrite in the culture supernatants were determined from a standard curve generated using sodium nitrite (NaNO_2_) standards provided in the Griess reagent kit (Invitrogen, Carlsbad, CA, USA) and expressed as µM nitrite.

### 4.9. Measurement of ROS Generation

Intracellular ROS generation was measured using the fluorescent probe 5-(and-6)-carboxy-2′,7′-dichlorofluorescein diacetate (CM-H_2_DCFDA), which is oxidized by ROS to produce a fluorescent signal. BV2 cells were seeded into 96-well plates and cultured under standard conditions. After 24 h of incubation, the cells’ media were removed and replaced with 10 μM CM-H_2_DCFDA in phosphate-buffered saline (PBS) and incubated for 20 min at 37 °C in a humidified incubator containing 5% CO_2_ under minimal light exposure to prevent photobleaching. The cells were then washed with PBS to remove excess dye. Subsequently, the cells were treated with 1 μg/mL LPS in the presence or absence of *B. thailandica* leaf extract at the indicated concentrations, or with quercetin (10 μM) as a positive control, in serum-free DMEM for 24 h. After treatment, fluorescence was measured using a BioTek Synergy H1 multimode microplate reader (BioTek Instruments, Winooski, VT, USA). The excitation wavelength was set at 488 nm, and the emission wavelength was 520 nm, corresponding to the fluorescence of 2′,7′-dichlorofluorescein (DCF), the oxidized product of CM-H_2_DCFDA. ROS levels were quantified by measuring DCF fluorescence, reflecting the degree of intracellular oxidative stress and the antioxidant activity of the *B. thailandica* leaf extract [54]. ROS levels were normalized to the LPS-treated group (set as 1).

### 4.10. Enzyme-Linked Immunosorbent Assay (ELISA)

After 24 h of treatment, the culture medium from each well was collected and centrifuged to remove cellular debris. The resulting cell culture supernatants were used for cytokine analysis. Prior to ELISA analysis, the collected supernatants were diluted with sample diluent as follows: IL-1β samples were diluted 1:2, IL-6 samples were diluted 1:6, while TNF-α samples were analyzed without dilution. The levels of IL-6, IL-1β, and TNF-α in the culture supernatants were quantified using commercial ELISA kits (R&D Systems, Minneapolis, MN, USA) according to the manufacturer’s instructions. Briefly, 50 µL of assay diluent was added to each well of the antibody-coated microplate, followed by 50 µL of either cytokine standards or prepared samples. The plates were covered and incubated at 37 °C for 2 h, then washed four times with washing buffer. Subsequently, the biotinylated detection antibody was added, and the plates were incubated at room temperature for an additional 2 h. After washing, substrate solution was added and incubated at room temperature for 30 min, and the reaction was stopped by adding the stop solution to each well. Absorbance was measured at 450 nm using a microplate reader (BioTek Instruments Inc., Winooski, VT, USA). Cytokine concentrations were calculated based on the standard curves generated from the corresponding cytokine standards. The final concentrations were obtained by multiplying the calculated values by the respective dilution factors and were expressed as cytokine levels (pg/mL) in the culture supernatant.

### 4.11. Statistical Analysis

Data are presented as the mean ± SEM. Multi-group comparisons were carried out using one-way ANOVA, followed by the Bonferroni post hoc test. Differences among the means were considered statistically significant at *p* < 0.05.

## 5. Conclusions

**Figure 7:**
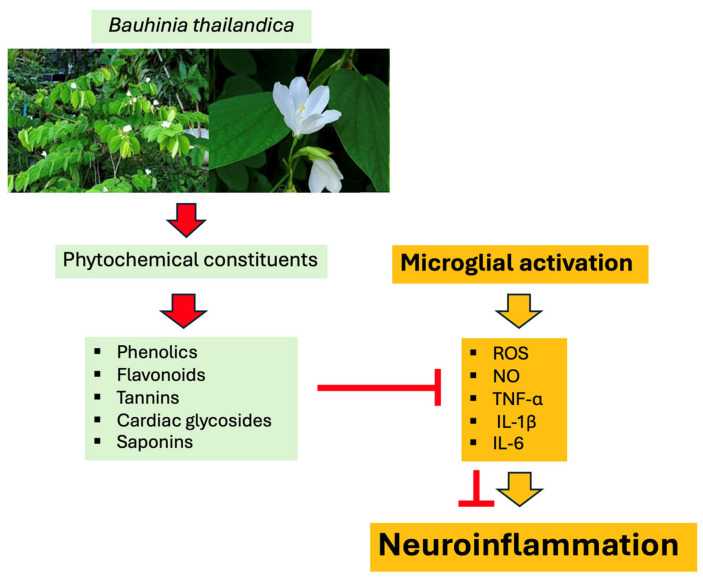
Phytochemical constituents of *B*. *thailandica* and their potential role in suppressing microglia-mediated neuroinflammation. Schematic representation of the phytochemical components of *B. thailandica* leaf extract, comprising phenolics, flavonoids, tannins, cardiac glycosides, and saponins, along with their prospective influence on microglial activation modulation. The extract inhibits the formation of intracellular ROS, nitric oxide (NO), and pro-inflammatory cytokines TNF-α, IL-1β, and IL-6 in LPS-stimulated BV2 microglial cells. These actions combined facilitate the reduction in microglia-mediated neuroinflammation and indicate a possible neuroprotective function of the leaf extract.

The present study presents the first evidence that *Bauhinia thailandica* leaf extract exhibits substantial antioxidant and anti-inflammatory properties in LPS-stimulated BV2 microglial cells. The extract did not exhibit any cytotoxicity within the concentration range that was tested. It substantially diminished the production of intracellular ROS and NO and the release of pro-inflammatory cytokines (TNF-α, IL-1β, and IL-6), which are critical mediators of neuroinflammation driven by microglia. The high levels of phytochemicals in the *B. thailandica* leaf extract, which include phenolics, flavonoids, tannins, cardiac glycosides, and saponins, are likely the cause of the observed biological activities (Figure 7). The present findings highlight the therapeutic potential of the leaf extract from *B. thailandica* as a natural source of bioactive compounds for the prevention of neuroinflammation. However, further research is required to isolate and characterize the individual active components and to clarify their specific molecular targets and signaling pathways. Phytochemical characterization through LC–MS/MS profiling or HPLC fingerprinting would specifically aid in identifying the predominant constituents and assist in the standardization of the extract. Furthermore, validation in in vivo models of neuroinflammatory and neurodegenerative disorders is necessary to establish its potential translational relevance.

## Figures and Tables

**Table 1:** Qualitative phytochemical screening of the leaf extract of *B*. *thailandica*.

Phytoconstituents	Test Method	*B. thailandica*
Phenolics	Ferric chloride test	+
Flavonoids	Ammonia test	+
Tannins	Ferric chloride test	+
Cardiac glycosides	Keller–Killiani test	+
Saponins	Frothing test	+
Terpenoids	Salkowski test	−
Steroids	Salkowski test	−
Alkaloids	Dragendorff’s test	−
Anthraquinones	Borntrager’s test	−

(+ = Present, − = Absent).

**Table 2:** Total phenolic, flavonoid, and tannin contents of the ethanolic leaf extract of *B*. *thailandica*.

Parameter Analyzed	Values Obtained
Total phenolic content (mg GAE/g dry extract)	70.55 ± 1.35
Total flavonoid content (mg QE/g dry extract)	249.47 ± 1.24
Total tannin content (mg TAE/g dry extract)	397.50 ± 4.44

Notes: GAE = gallic acid equivalent; QE = quercetin equivalent; TAE = tannic acid equivalent. Values are presented as mean ± SD.

**Table 3:** DPPH radical scavenging activity of *B. thailandica*.

Sample	Test Concentration (µg/mL)	% Inhibition	IC_50_ Value (µg/mL)
*B. thailandica*	1000	89.89 ± 1.36	513.60 ± 7.20
Vitamin C	100	80.91 ± 0.42	68.25 ± 2.31

## Data Availability

The original contributions presented in this study are included in the article. Further inquiries can be directed to the corresponding author.
